# Nonocclusive mesenteric ischemia during treatment for ketoacidosis associated with acute‐onset type 1 diabetes mellitus: A case report

**DOI:** 10.1002/ccr3.5714

**Published:** 2022-04-20

**Authors:** Kazunobu Une, Yusuke Sumi, Manabu Kurayoshi, Ryuichi Nakanuno, Masahiro Nakahara

**Affiliations:** ^1^ 46664 Department of Emergency Medicine JA Onomichi General Hospital Onomichi City Japan; ^2^ 46664 Department of Surgery JA Onomichi General Hospital Onomichi City Japan; ^3^ 46664 Department of Anesthesiology JA Onomichi General Hospital Onomichi City Japan

**Keywords:** acute‐onset type I diabetes, anti‐GAD antibody diabetic ketoacidosis, laparoscopy, nonocclusive mesenteric ischemia

## Abstract

This case report describes a patient with nonocclusive mesenteric ischemia that developed due to diabetic ketoacidosis. We believe that early diagnosis and intervention may improve the prognosis of nonocclusive mesenteric ischemia that has low vascular risk, with the major risk factor being dehydration due to diabetic ketoacidosis.

## Graphical abstract

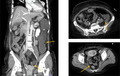

## 1 INTRODUCTION

Nonocclusive mesenteric ischemia (NOMI) was first reported by Ende et al.^1^ in 1958 as a syndrome of small intestinal necrosis in patients with heart failure. Subsequently, hypoperfusion of the intestines due to various factors induces mesenteric vascular spasm. Even with no obstruction in the main mesenteric blood vessels, widespread discontinuous intestinal blood flow disorders in a segmental manner may still occur.^2^ Among acute mesenteric insufficiency diseases, the incidence rate of NOMI is relatively high at 15%–27% in Japan, with a case fatality rate of 50–80%, which warrants poor prognosis.^3^ We encountered a case of NOMI suspected to be complicated by intestinal hypoperfusion during the treatment of diabetic ketoacidosis (DKA). Appropriate surgical intervention was performed to save the patient's life.

No consent was obtained from the Ethics Committee as it is not necessary for case reports. Furthermore, the data were anonymized based on the Personal Information Protection Law, and consent from the patient, their family, or a similar guardian was obtained for publication of the case report. The authors declare no conflict of interest.

## 2 CASE PRESENTATION

The patient was a 54‐year‐old woman.

[Previous diseases]

Monthly medication for hyperlipidemia

[Symptoms/physical findings at emergency department]

Height, 161 cm; weight, 64 kg; body mass index, 24.7 kg/m^2^.

GCS, 11 (E2V4M5); blood pressure, 60/20 mm Hg; heart rate (HR), 110 bpm; respiratory rate, 30 breaths/min; and body (bladder) temperature, 34℃.

The patient had tenderness in the upper abdomen.

The results of blood and urine examinations are shown in Table 1.

**TABLE 1:** Blood examination and urinalysis on admission

Complete blood count			Chemical test		
WBC	6750	/μl	TP	7.2	g/dl
Ne%	90.2	%	Alb	4.2	g/dl
RBC	539	×10^4^/μl	AST	19	IU/L
Hb	16.6	g/dl	ALT	26	IU/L
Hct	56.8	%	LDH	262	IU/L
PLT	21.1	×10^4^/mm^3^	CK	263	IU/L
Coagulation test			BUN	75.9	mg/dl
PT	>130	%	Cr	4.07	mg/dl
APTT	25.1	s	Na	137	mEq/L
Fib	307	mg/dl	K	5.3	mEq/L
D‐dimer	49.1	μg/ml	Cl	99	mEq/L
Blood gas analysis			CRP	2.11	mg/dl
pH	6.887	mg/dl	HbA1c	13.7	%
po2	107.0	mm Hg	Urine test		
pco2	34.6	mm Hg	Protein	2+	
HCO3	6.2	mEq/L	Sugar	4+	
BE	−29.7	mmol/L	Ketone body	2+	
Glucose	1654	mg/dl	Diabetes‐related test		
Lactate	3.7	mmol/L	GAD antibody	>2000	IU/ml
			CPR	0.46	ng/ml

### 2.1 Clinical course

**FIGURE 1:**
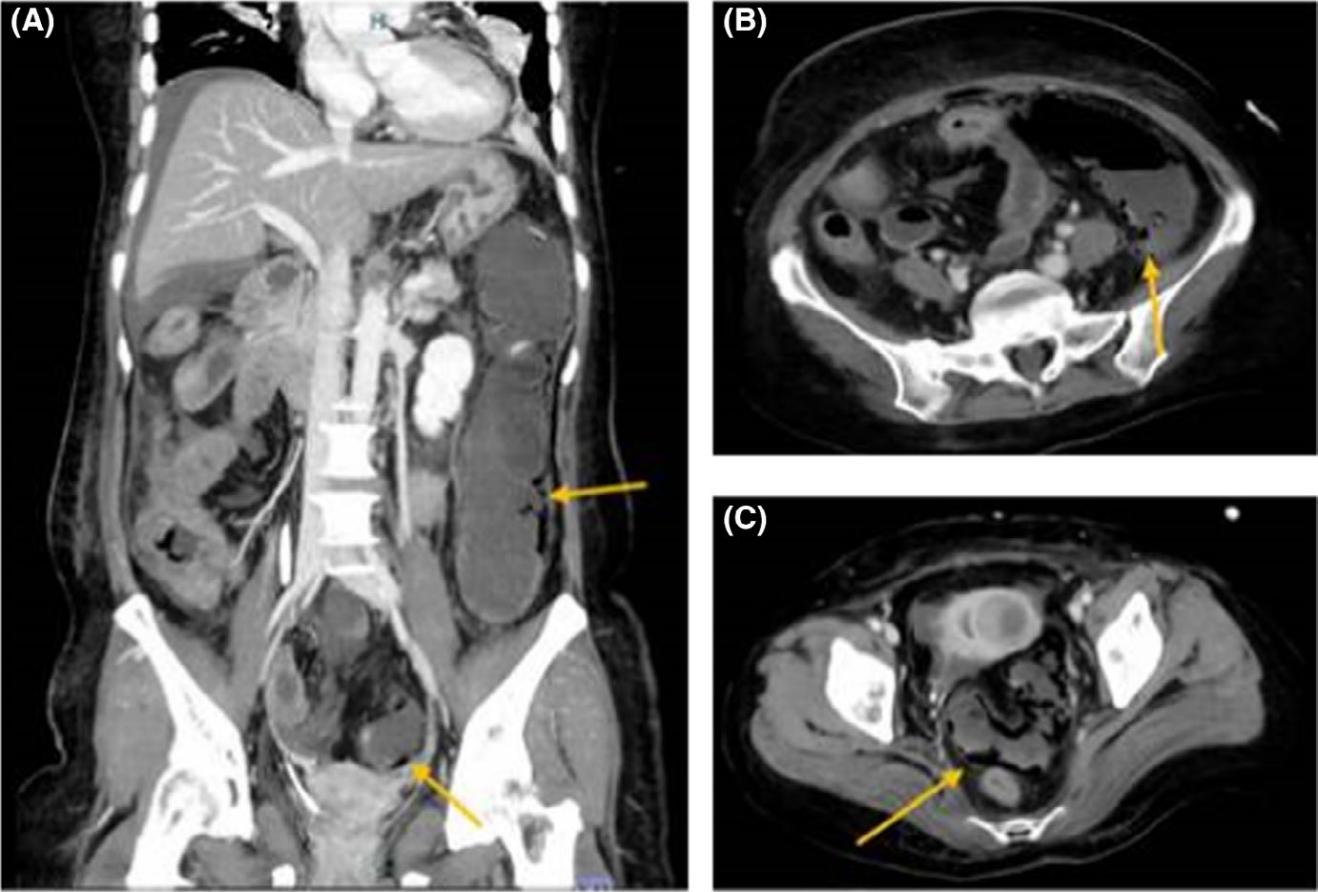
Abdominal CT on the 4th day. (A) Sagittal image. (B, C) Coronal section. Pneumatosis intestinalis is observed in the descending and sigmoid colon

The patient complained of persistent fatigue with repeated vomiting for 3 days prior to the consultation. The patient had black vomitus and a decreasing level of consciousness that prompted the transfer to our hospital by ambulance. Arterial blood gas showed a pH of 6.887, base excess of 29.7, and anion gap of 59. Urinalysis showed levels of ketone at 3+ and blood glucose at 1300 mg/dl. Treatment was initiated to address DKA‐associated shock. Immediate fluid resuscitation, insulin administration, and mechanical ventilation commenced. Noradrenaline was continuously administered at a maximum dose of 0.30 μg/kg/min to maintain a mean arterial pressure of 75 mm Hg or higher. Twenty‐four hours after admission, 1200 ml of urine outflow was observed for 7000 ml of fluid replacement, but tachycardia continued. Although the dose could be reduced following adequate fluid resuscitation, we could not reduce it below 0.10 μg/kg/min. Although consciousness improved to GCS 10T (E3VTM6), laboratory data remained poor, and the inflammatory response was prolonged. On the third day, myogenic enzyme levels were elevated, and abdominal ultrasonography was performed. Despite the presence of changes in the intestinal edema, ascites was not observed. Abdominal pain as a subjective symptom was unclear. On the fourth day, myogenic enzyme levels continuously increased, prompting an evaluation using contrast‐enhanced computed tomography (CT). CT revealed ascites around the spleen and liver and pneumatosis intestinalis in the left colon (Figure 1).

## 3 INVESTIGATIONS AND TREATMENT

**FIGURE 2:**
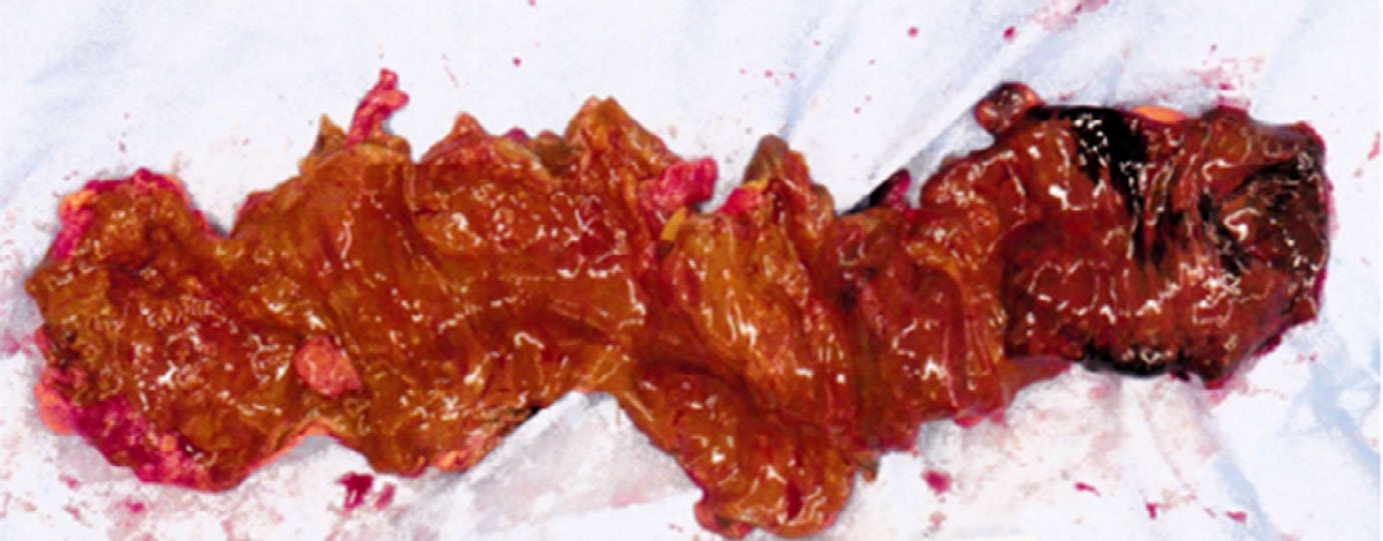
Resected intestine, whose length is approximately 45 cm. On the mucosal surface, a patchy ischemic area is observed on the anal side

Intestinal ischemia was considered, but the extent of ischemia was difficult to determine; thus, intraperitoneal observation with a laparoscope was performed immediately. Macroscopic findings showed a wide range of purple tones in the left colon, especially from the transverse to the sigmoid colon (Figure 2). In contrast, the color tone of the mesentery was good. We decided that extensive intestinal resection was necessary, and laparotomy was performed. No obstruction in the superior and inferior mesenteric arteries was observed; therefore, nonocclusive intestinal ischemia was diagnosed. In addition to left hemicolectomy and anterior rectal resection, transverse colon stoma construction was performed. Changes in edema were strong in the small intestine, and color changes and thin walls were observed; however, an excision was deemed unnecessary. The postoperative course was uneventful. The patient was extubated 2 days after surgery (the seventh day) and was discharged from the intensive care unit on the 13th day. Serum lactate levels are often used as an indicator of tissue ischemia. In this case, blood lactate level was 3.7 mmol/L at admission. After admission, it decreased to 1.5 mmol/L due to fluid resuscitation, but increased again on the 4th day, reaching a maximum of 4.1 mmol/L before surgery. After the operation, it rapidly decreased to 1.1 mmol/L, and no increase was observed thereafter. During the clinical course, a positive anti‐glutamic acid decarboxylase antibody was detected, while blood C‐peptide immunoreactivity was low (Table 1). The diagnosis of type I diabetes was made due to the results of anti‐glutamic acid decarboxylase antibody and the decrease in insulin secretory capacity. Glycated hemoglobin level was elevated on arrival, but it did not meet the diagnostic criteria for fulminant type 1 diabetes. A detailed clinical course was unknown because the patient had not been treated before for diabetes, and there were no data before admission. We considered that onset was with ketoacidosis; thus, we diagnosed it as acute‐onset type I diabetes. The patient was diagnosed with rapidly progressive type 1 diabetes. Subsequently, the blood glucose level was controlled by insulin administration, and the patient was discharged on the 63rd day.

## 4 DISCUSSION

When hypoperfusion lasts for 2 or more hours, blood vessels contract and the sympathetic nerves in the periphery of the mesenteric artery react excessively, causing intestinal ischemia and irregular spasms of the blood vessels.^4^ Although our patient was relatively young and had low vascular risk, hypoperfusion occurred due to frequent vomiting and dehydration owing to marked hyperglycemia by DKA. Black vomitus was considered temporary bleeding due to repeated vomiting. After the patient's arrival at our hospital, vomiting was not observed, and hemodynamics were unstable; thus, upper gastrointestinal endoscopy was not performed. No black vomitus was seen from the indwelling nasogastric tube, and it is considered that NOMI was not present at the start of treatment, as shown by the imaging findings at admission.

The gold standard for diagnosing NOMI is angiography. However, there is often insufficient time to perform it. In such cases, multidetector CT (MDCT) has been reported useful in early diagnosis.^5^ In our case, whole‐body CT screening on admission was unremarkable, but a repeat CT was performed after no improvement in tachycardia and elevated myogenic enzyme levels. Although the onset time was unknown, the repeat CT showed pneumatosis intestinalis and ascites retention approximately 70 h after admission, leading to the suspicion of NOMI. If intestinal necrosis is suspected, surgical treatment should be promptly performed. It has been reported that the survival rate is lower if the time between onset and the start of treatment exceeds 24 h compared with intervention initiated within 24 h (58.7% vs. 84.6%).^6^ It is also necessary to select a diagnostic method that is also an intervention such as laparoscopy without delay if the diagnosis is uncertain or if the extent of ischemia is difficult to determine. For patients who have underlying diseases with high vascular risk, such as heart failure, or those on dialysis, it is highly possible that the vascular properties are associated with a poor prognosis. Kamatani et al. reported that of nine patients with NOMI triggered by hypoperfusion associated with DKA, eight (88.9%) recovered.^7^ In cases of juvenile‐onset or sudden hypoperfusion due to DKA, there is a high possibility that multidisciplinary treatments, such as infusion and insulin administration, can improve hemodynamics at an early stage. Early diagnosis and intervention are also warranted. These ultimately lead to a good prognosis.

## 5 CONCLUSIONS

Although NOMI is a disease with a poor prognosis, it is possible that it has low vascular risk, with the main risk factor being dehydration such as DKA, which has good therapeutic responsiveness. In addition, since early diagnosis is required, rapid diagnostic imaging using MDCT and aggressive diagnostic laparoscopy appears useful.

## CONFLICT OF INTEREST

None.

## AUTHOR CONTRIBUTIONS

Kazunobu Une and Ryuichi Nakanuno provided clinical care for the patient. Yusuke Sumi, Manabu Kurayoshi, and Masahiro Nakahara performed the operation. Kazunobu Une contributed to the conception, literature review, and drafting. All authors read and approved the final manuscript.

## APPROVAL OF THE RESEARCH PROTOCOL

N/A.

## CONSENT

Written informed consent was obtained from the patient for publication of this case report and any accompanying images.

## REGISTRY AND REGISTRATION OF THE STUDY/TRIAL

N/A.

## ANIMAL STUDIES

N/A.

## Data Availability

The data that support the findings of this study are openly available.
